# The impact of climate change on vulnerable populations in pediatrics: opportunities for AI, digital health, and beyond—a scoping review and selected case studies

**DOI:** 10.1038/s41390-024-03719-x

**Published:** 2025-01-29

**Authors:** Elizabeth A. Campbell, Felix Holl, Harleen K. Marwah, Hamish S. Fraser, Sansanee S. Craig

**Affiliations:** 1 Department of Environmental Health and Engineering, Johns Hopkins Bloomberg School of Public Health, 615 N. Wolfe St., Baltimore, MD 21205 USA; 2 Center for Outbreak Response Innovation, Johns Hopkins Bloomberg School of Public Health, 700 E. Pratt Street, Suite 900, Baltimore, MD 21202 USA; 3 Department of Biomedical Informatics, Columbia University Irving Medical Center, 622 W 168th St PH20 3720, New York, NY 10032 USA; 4 DigiHealth Institute, Neu-Ulm University of Applied Sciences, Neu-Ulm, Germany; 5 Division of General Pediatrics, The Children’s Hospital of Philadelphia, Philadelphia, PA USA; 6 Brown Center for Biomedical Informatics, The Warren Alpert Medical School of Brown University, Providence, RI USA; 7 Division of General Pediatrics, Department of Pediatrics, The Children’s Hospital of Philadelphia, University of Pennsylvania, Perelman School of Medicine, Philadelphia, PA USA

## Abstract

**Abstract:**

Climate change critically impacts global pediatric health, presenting unique and escalating challenges due to children’s inherent vulnerabilities and ongoing physiological development. This scoping review intricately intertwines the spheres of climate change, pediatric health, and Artificial Intelligence (AI), with a goal to elucidate the potential of AI and digital health in mitigating the adverse child health outcomes induced by environmental alterations, especially in Low- and Middle-Income Countries (LMICs). A notable gap is uncovered: literature directly correlating AI interventions with climate change-impacted pediatric health is scant, even though substantial research exists at the confluence of AI and health, and health and climate change respectively. We present three case studies about AI’s promise in addressing pediatric health issues exacerbated by climate change. The review spotlights substantial obstacles, including technical, ethical, equitable, privacy, and data security challenges in AI applications for pediatric health, necessitating in-depth, future-focused research. Engaging with the intricate nexus of climate change, pediatric health, and AI, this work underpins future explorations into leveraging AI to navigate and neutralize the burgeoning impact of climate change on pediatric health outcomes.

**Impact:**

- Our scoping review highlights the scarcity of literature directly correlating AI interventions with climate change-impacted pediatric health that disproportionately affects vulnerable populations, even though substantial research exists at the confluence of AI and health, and health and climate change respectively.
- We present three case studies about AI’s promise in addressing pediatric health issues exacerbated by climate change.
- The review spotlights substantial obstacles, including technical, ethical, equitable, privacy, and data security challenges in AI applications for pediatric health, necessitating in-depth, future-focused research.

## Introduction

### Climate change and pediatric health

Anthropogenic climate change is a significant challenge to the global community that is already impacting population and clinical health outcomes throughout the world.^1,2^ Its impact on human health and environmental well-being is only expected to grow over the next century as climate change’s effects on societies become more acute.^3^ Climate change results in higher temperatures globally, changes to precipitation patterns (greater precipitation in some areas and drought in others), increased adverse weather events and rising sea levels.^2,4^ In turn, these changes to weather patterns negatively impact agricultural production, decrease productivity, and displace communities living in these areas. Deleterious effects on human health include heat stress, vector-borne disease spread, food insecurity, malnutrition, increased prevalence of allergic and respiratory issues, homelessness and loss of income, and worsened mental health.^2^ These negative consequences disproportionately impact vulnerable populations, namely those living in low-income countries and the resource-denied in high-income countries.^4,5^

Children have been identified as among the most vulnerable subpopulations to these health consequences, in part because of their developing physiologies (including respiratory and immune systems) and the long-term exposure to climate change-induced societal impacts that they will face over their life course.^2,6,7^ Children have less physical strength, may become separated from parents during weather-related natural disasters, and are particularly vulnerable to the health risks of higher temperatures and of flood waters due to risk of drowning or waterborne disease.^2,4^ Climate change worsens pediatric asthma and allergies, and leads to mental and physical traumas from disasters and climate-related anxieties about the future.^8,9^ Finally, children are especially vulnerable to the effects of malnutrition, vector-borne diseases, and a lack of access to food and clean water that climate change is increasing the prevalence of.^4,8^

### AI, digital health, and climate change

While the current and future impacts of climate change to child health are significant, tools and strategies exist to help mitigate these. Two promising and related areas are Digital Health systems and Artificial Intelligence (AI). In this paper, we define digital health as a collection of electronic tools, such as telemedicine, wearable devices, electronic medical records, and mHealth, and their appropriate application to promote improved individual and population health outcomes.^10^ These systems have been developed and implemented in LMICs over more than 2 decades and are an increasingly important part of healthcare delivery, public health and surveillance, supply chain management and disaster response around the world.^11^ An increasing body of research supports the beneficial effects of digital health interventions for these uses with examples from routine care of chronic diseases like HIV/AIDS^12^, Tuberculosis (TB)^13^ and maternal and child health care^14^, pediatric urgent care^15^ and the Ebola and COVID outbreaks.^16–19^ Digital health systems developed and used in LMICs are usually adapted to infrastructure challenges like lack of stable electric power, limited internet connectivity, limited user training, and the need for robust and low-cost devices. These characteristics can allow their use during a variety of natural and man-made disasters.

We define AI as a branch of computer science that aims to build intelligent tools that represent aspects of the human mind and can complete intellectual tasks that humans can perform.^20,21^ AI subfields that may also be used together in a single application include machine learning, expert systems, natural language processing, and image and signal processing.^22^

AI may be used in myriad ways to enhance humans’ understanding of climate change and combat its societal impacts.^23^ AI’s ability to analyze large and complex datasets is crucial to information systems for surveilling and predicting climate trends, extreme weather events, epidemics, etc.^5,24^ Advances in AI are complementary to developments in digital health that may help to alleviate climate change’s human impacts. Digital health interventions such as telemedicine and personalized health monitoring expand healthcare access for vulnerable, geographically remote, and/or underserved communities, who are often the most impacted by climate change’s health impacts. In turn, AI can support improved diagnosis, risk assessment, and disease outbreak prediction to support digital health applications. AI applications can also be trained on data from Digital Health applications, and in turn be deployed as part of decision support tools in such systems.^22,25^

### Objectives and scope of the scoping review

In this work, we present findings from a scoping review to assess current literature on AI applications for addressing health effects of climate change on pediatric health. We developed our search and review strategies along with our inclusion and exclusion criteria in accordance with the Preferred Reporting Items for Systematic Reviews and Meta-Analysis (PRISMA) Extension for Scoping Reviews guidelines.^26^ This review helps to contextualize current literature on how AI and other health information technologies may be used to address climate-change-induced population and clinical pediatric health outcomes, ethical, and equitable considerations for using AI in pediatric healthcare, and gaps for future research into how AI may be used to offset negative implications of climate change on pediatric health.

## Methods

Using the MeSH database^27^ as a starting point, we developed a keyword list to represent key concepts for the scoping review, namely “Artificial Intelligence”, “Child Health”, and “Climate Change” in the PubMed Database. The keyword search was iteratively refined through team discussion and consultation with an experienced librarian at the Children’s Hospital of Philadelphia until the number of resulting articles approached reviewer feasibility. (Table 1, Final Query in Appendix). Reviewers together screened a representative sample of initial results, discussed, and amended the keylist until no additional keyword changes yielded relevant articles and saturation was felt to have been reached. Reviewers also hand searched the reference lists of relevant articles to find relevant previous work as well as checked the citations of those articles to catch more recently published work. All reviewers reviewed the final selected articles together. The scoping review was conducted using keyword searches of all literature published from January 1, 2007- June 30, 2023 in PubMed, EMBASE, and IEEE Xplore. We selected the January 2007 cutoff to align with major climate change policy milestones that began to inform modern scientific understanding and research on climate change (including the Kyoto Protocol’s ratification in 2005 and the Intergovernmental Panel on Climate Change’s 2007 Synthesis Report), as well as an observed trend in climate change publications on PubMed, starting with a significant and sustained increase beginning in 2007. Articles identified in the initial database scan were retained for the review based on the following inclusion and exclusion criteria:

**Table 1:** AI Applications in Climate Change Effects on Pediatric Health Scoping Review Keywords.

Artificial Intelligence	“Intelligence, Artificial” OR “Machine Intelligence” OR “Intelligence, Machine” OR “Artificial Intelligence” OR “Machine Learning” OR “Deep Learning” OR “Supervised Machine Learning” OR “Support Vector Machine” OR “Unsupervised Machine Learning” OR “Computer Heuristics” OR “Expert Systems” OR “Fuzzy Logic” OR “Natural Language Processing” OR “Neural Networks, Computer” OR “Robotics” OR “Sentiment Analysis”	AND
Child Health	“Pediatric Medicine” OR “Child Services, Health” OR “Health Services, Child” OR “Child Health Service” OR “Health Service, Child” OR “Service, Child Health” OR “Services, Child Health” OR “Infant Health Services” OR “Infant Services, Health” OR “Health Services, Infant” OR “Health Service, Infant” OR “Infant Health Service” OR “Service, Infant Health” OR “Services, Infant Health” OR “Pediatrics” OR “Adolescent Medicine” OR “Pediatric Emergency Medicine” OR “Child” OR “Child Health” OR “Health, Child” OR “Children’s Health” OR “Health, Children’s” OR “Child Well Being” OR “Well Being, Child” OR “Well-Being, Child” OR “Child Wellbeing” OR “Wellbeing, Child”	AND
Climate Change	“Climate Change” OR “Change, Climate” OR “Changes, Climate” OR “Climate Changes”	

### Inclusion criteria

- Peer-reviewed full publications and conference proceedings
- Empirical studies or Review Papers
- Focus on studying the health impacts of climate change
- Must include discussion of climate change impacts to pediatric health (but does not have to be exclusively focused on child health)
- Includes a discussion on utilizing artificial intelligence and other information technologies to assess or mitigate population and clinical health impacts of climate change
- May include studies utilizing quantitative, qualitative, or mixed methods approaches to data analysis

### Exclusion criteria

- Conference abstracts, posters, and book chapters
- Not English language
- Published before 2007

As this study does not include any human subjects research, we did not require approval from an Institutional Review Board (IRB).

## Results

An initial query of PubMed using keywords for climate change and AI and health resulted in an infeasible >46,131 results. Limiting this to “child health”, “climate change”, and “artificial intelligence” or “machine learning” as MeSH major topics, subheadings or terms yielded only 2 results, neither of which met inclusion criteria. After keyword iteration, the final query yielded 11 articles, of which only 2 met inclusion criteria. The two articles that met our inclusion criteria are included in Table 2. Querying additional databases (EMBASE and IEEE) yielded no additional studies.

**Table 2:** Studies meeting the inclusion criteria for evaluating AI solutions to addressing child health impact from climate change.

Article	Child Health Condition	Climate Change Effect	AI Solution
Differential respiratory health effects from the 2008 northern California wildfires: A spatiotemporal approach. Reid CE, et al. Environ Res. 2016 Oct	Pediatric asthma hospitalizations and ED visits, triggered by air pollution	Air particulates from increasing wildfires due to temperature changes	Data-adaptive machine learning methods from a large set of spatiotemporal data set to find evidence of differential susceptibility to exposure to wildfire smoke based on age
Deciphering the black box of Omics approaches and artificial intelligence in food waste transformation and mitigation. Sharma P, et al. Int J Food Microbiol. 2022 Jul	Pediatric malnutrition/starvation	Food waste contributes to increased greenhouse gases that contribute to climate change	Omics and artificial intelligence to reduce wastage of food at various stages of the food cycle

**Fig. 1:**
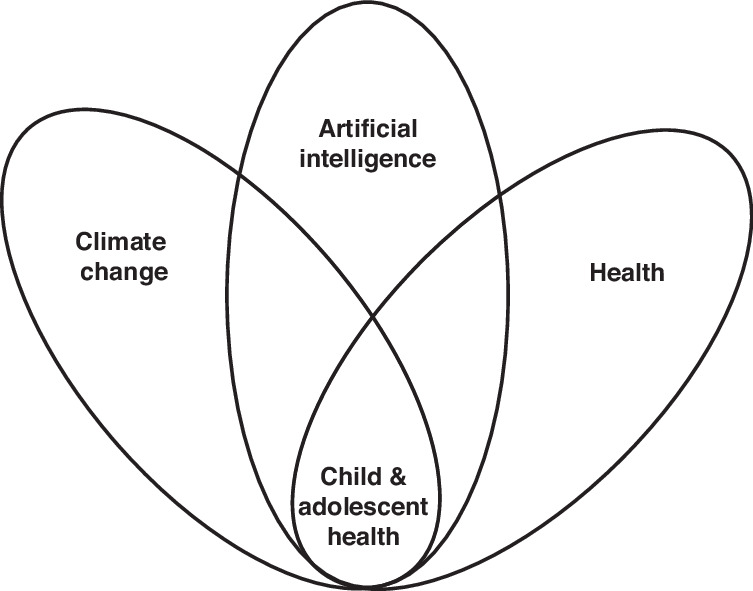
Emerging Themes from Scoping Literature Review on AI, Climate Change, and Child Health. While there is a wealth of literature at the individual intersections of Artificial Intelligence, Health, and Climate Change, research specifically examining the nexus of these three domains in child and adolescent health outcomes remains limited.

Major themes emerged in our scoping literature review. Large bodies of literature exist at the intersection of AI/Digital Health and Health (not pediatric specific) and climate change (>46,000 results) OR child health and climate change (411 results), but only two at the nexus of all three. (see Fig. 1). We did find articles that addressed clinical problems in children known to be increasing in relation to global heating and climate change but did not directly address impact on child health. These include water-borne diseases, vector-borne diseases, effects of disruptions including trauma to living conditions and housing, forced migrations, malnutrition, asthma and allergies. One systematic review noted major gaps in climate health research for child health, and none have addressed the use of AI for combating pediatric health conditions worsened by climate change.^28^ If AI solutions were mentioned, most articles vaguely included this concept in the discussions section as a potential future tool, without specific details and none specifically focused on pediatric health.

Given the gap in current literature addressing this topic, authors identified articles through lived experience and subject matter expertise in global health informatics that best illustrated the potential for AI and digital health solutions to address the key overlaps between pediatric health and climate change impacts.

### AI solutions for child health conditions related to climate change

#### Case 1: AI and pediatric diarrhea due to flooding and disrupted access to clean water

##### Child health condition

Diarrheal disease is a leading cause of morbidity and mortality of children in LMICs. The most important risk factor is unsafe drinking water which can be exacerbated by floods, storm surges, drought, and forced migration.

##### AI solution

Nelson and colleagues developed a prediction model for viral versus bacterial causes of diarrhea in children in low-income countries, a condition likely to increase in incidence due to climate change.^29^ The model was implemented in a smartphone application that indicated the probability of viral etiology based on patient and location characteristics. They evaluated the clinical impact of this mHealth tool in randomized cross over trials in both Bangladesh and Mali, with the primary outcome of the proportion of children prescribed an antibiotic. Both arms of the study were negative based on the primary endpoint. However, detailed analysis of the outputs of the model showed that the high predicted probability of viral etiology was linked to low antibiotic prescribing rates. This suggests that further studies would be beneficial on effects of machine learning outputs on clinical decision making.

A study by Afshan et al. from Punjab Province in Pakistan emphasized the changes in prevalence of Fasciolosis or liver fluke likely driven by changes in climate.^30^ They stress a particular risk to low lying, irrigated areas that were subsequently affected by severe floods in 2022. This GIS based approach has important potential for risk estimation and control of waterborne disease in response to impacts of climate change, as well as being based on the One Health paradigm.^31,32^ Another example is models that can guide the need for pre-positioning of critical supplies and medications in some African countries prior to the rainy season.

#### Case 2: AI and pediatric asthma exacerbations due to increasing air pollution

##### Child health condition

Climate change increases known asthma triggers, including extreme heat, more severe pollen seasons, wildfire smoke, and mold exposures.^33^ Additionally, research on air quality demonstrates that people of color are exposed to air pollution at disproportionately higher rates, further exacerbating health disparities.^34^ With 6.5% of children in the United States impacted by asthma in 2021, it is important to recognize these triggers and how climate change is impacting them.^35^

##### AI solution

AI has the potential to employ predictive modeling to guide anticipatory guidance and health system response to known asthma triggers. Currently, the Air Quality Index provides an air quality score based on ambient air quality and pollutants, including ground-level ozone, particulate matter (PM 2.5 and PM 10), carbon monoxide, sulfur dioxide, and nitrogen dioxide. The ultimate score is then correlated with different levels of health sensitivity that allows people to assess their health risks.^36^ Using predictive modeling and AI, other known drivers of asthma exacerbation like extreme heat and pollen, can be layered into the score to provide more detailed risk analysis. Further, in collaboration with health systems like emergency departments, urgent care centers, and primary care centers, data can be fed back into the system to continue to improve predictive accuracy and reliability. Ultimately, this may provide a beneficial adaptation strategy in the wake of climate change. On a patient level, people can rely on this system to assess personal risk. On a community level, schools can use this tool to drive decisions around outdoor play and safe activities. On a health system level, staffing and medications can be appropriately ramped up in higher risk times and areas. This model may have additional application if expanded to the heat-index to protect patients from heat-related illness.

#### Case 3: Digital health and AI to help control epidemics and pandemics

##### Child health condition

Both the 2014–2015 Ebola epidemic and the COVID-19 pandemic came with severe pediatric clinical problems. The Ebola epidemic involved children experiencing severe, often fatal, hemorrhagic fevers, given that children often have less developed immune systems than adults, managing and treating their Ebola infections presented unique challenges and often required intensive care and isolation to prevent further spread.^37^ During the COVID-19 pandemic, children generally experienced milder symptoms compared to adults, yet cases of Multisystem Inflammatory Syndrome in Children, a condition where multiple body parts can become inflamed, emerged as a significant concern.^38,39^

##### Digital health solutions

During the 2014–2015 Ebola epidemic, and also more recently during the COVID-19 pandemic, mHealth has demonstrated substantial utility in combating epidemics. Several mobile apps were developed and deployed with the aim to manage and contain the spread of these viruses. Some applications were specifically designed for contact tracing, which involves identifying and managing people who have been exposed to a disease to prevent its further spread.^40,41^ Some tools already included AI components, and all create datasets needed for future AI applications. Additional solutions include:

- Contact Tracing: Applications were used to systematically collect data about individuals who had come into contact with infected persons. These apps helped in tracking and managing individuals who were potentially exposed to Ebola to ensure they were monitored for symptoms and could be treated promptly if needed. One example mHealth platform CommCare was subsequently used in many countries to support contract tracing for COVID-19, including in the US state of Massachusetts.
- Surveillance and Data Management: Mobile apps as well as the District Health Information System (DHIS2) also facilitated the real-time reporting and management of data related to the spread of the epidemic. Health workers could report new cases, manage patient data, and share information promptly with relevant authorities and organizations. An additional application developed for the Ebola outbreak (by two members of the Helmholtz Association of German Research Centers) and subsequently used in many countries for disease surveillance is Surveillance and Outbreak Management Systems (SORMAS).^42^
- Communication: mHealth also provided a platform for the dissemination of accurate information to communities, promoting awareness and guiding people on precautionary and preventive measures.
- Resource Allocation: Mobile applications assisted authorities in monitoring the situation on the ground, enabling them to make informed decisions about where to allocate resources, such as medical staff, supplies, and treatment centers.
- Acute management of Ebola in Ebola Treatment Centers (ETC): Several EHR systems and mobile health applications were developed or adapted to support acute care. For example, the OpenMRS EHR was rapidly adapted and used in Kerrytown, Sierra Leone by Save the Children, and in Medicines Sans Frontiers supported Ebola treatment centers by staff of Google Inc.^17^

## Discussion

Our scoping review yielded few studies on how AI and digital health may be used to address climate change’s effects on pediatric health outcomes. The case studies described here were not identified in the scoping review likely due to challenges of common terminologies and keywords used across studies at this nexus of multiple disciplines, causing some studies to appear out of scope. In addition, machine learning initiatives in LMICs are just beginning to be published, as evidenced in other global scoping reviews on machine learning and health care disparity perpetuation.^43,44^ While AI (including machine learning) has many ongoing and potential uses to address climate change and support digital health interventions, its deployment and scale-up in resource-poor settings is still in its early stages, and its applications to address the specific impacts that climate change has on pediatric health remains understudied.^22^ As this area of study evolves, there are numerous ethical challenges and considerations that need to be taken to deploy AI effectively.

### Challenges and ethical considerations

From a technical standpoint, common challenges to AI in medicine include overfitting and underfitting during machine learning model training. Overfitting occurs when a model becomes too specialized to the training data, e.g., data from patients with a known diagnosis who are from a particular group or location, performing well on the test data set but failing to generalize to new data. Underfitting happens when a model is too simplistic and cannot capture the underlying patterns in the data.^45^ Both challenges limit the utility of AI models. Digital health tools and AI applications in particular are also very sensitive to the quality and representativeness of the clinical data collected and analyzed. In an applied context, AI also raises concerns surrounding privacy, data security, and environmental impact.

### Environmental impact of AI itself

Training AI models requires a large amount of computational power and massive amounts of electricity, which in turn increases energy demand and the fossil fuels needed to generate this power.^46^ The data centers where large-scale AI models are trained and deployed also are “thirsty” and require large amounts of water to support cooling systems to ensure that servers don’t overheat.^47^ Finally, expanded AI use has increased the demand for hardware and the raw materials to produce it, whose extraction negatively impacts the planet’s air and soil quality.^48,49^ Although AI has many potential uses in tackling environmental and health issues that stem from a rapidly changing climate, its own carbon footprint must be considered in parallel with the benefits it provides. Critically evaluating and actively mitigating AI’s own climate impacts is part of implementing ethical and responsible technological implementation to fully realize AI’s potential to fight climate change and promote pediatric health outcomes.^50^

### Privacy and data security issues associated with AI solutions

The use of AI in healthcare often requires the collection and analysis of large volumes of sensitive patient data. Major concerns associated with using AI solutions in healthcare are data access, use, and control.^51^ Ethical concerns arise when there is inadequate protection of patient privacy and insufficient safeguards to prevent data breaches or unauthorized access. Patients must have confidence that their personal health information is being handled securely. Informed consent to use data in AI applications must be obtained but rarely is, even for the data used to train and test the models.^52^

### Bias and fairness in AI algorithms for pediatric health care and decision-making

An important goal when using AI to address pediatric health issues is to ensure algorithmic fairness and minimize potential biases. AI systems are trained on vast amounts of data based on previous clinical and health system activity, which can inadvertently contain biases. If these biases are not adequately addressed, AI algorithms can perpetuate or amplify existing healthcare disparities, resulting in unfair treatment across different demographic groups.

Bias could occur if the data used for training is not representative of the entire pediatric population or if it reflects existing healthcare disparities. For example, if the algorithm is trained primarily on data from specific demographics or geographic regions, it may not provide accurate recommendations for children from underrepresented groups.^52^ That said, GIS data is acknowledged to provide a foundation for modeling and AI methods, including climate changes in pediatric asthma, but was not included as a keyword in this study. Pediatric healthcare often requires considering the child’s specific context, including their family environment, cultural background, and social determinants of health. AI algorithms must account for these contextual factors to provide appropriate and culturally sensitive recommendations. Ignoring these factors can result in biased or ineffective care.^53^

Age-related bias is another concern for AI model development to address pediatric health issues. Pediatric healthcare involves children of different ages, from newborns to adolescents. AI algorithms should account for age-related differences in health conditions, treatments, and outcomes. Failing to address these differences can lead to inaccurate predictions or recommendations.^54^

Finally, AI algorithms often work as black boxes, making it difficult for healthcare professionals and patients to understand how a specific decision was reached. Lack of transparency and explainability can undermine trust and raise concerns about accountability, especially when critical healthcare decisions are involved. Measures need to be taken to ensure safety and transparency.^52^ Healthcare providers and parents should be able to understand and interpret the recommendations made by the AI system. This promotes trust and allows for critical evaluation and intervention when necessary, and may increase the likelihood that users follow potentially valuable recommendations from an AI based system.^55^

### Implementation considerations of AI into clinical workflows

AI’s integration into clinical workflows may have unintended consequences, including provider burnout, safety, and unclear impacts on clinical outcomes. AI has the potential to improve clinician well-being by supporting personalized continuing medical education to keep clinicians apprised of advancements to their fields and changes in patient recommendations, as well as being integrated into clinicians’ clinical documentation and quality measurement tasks.^56^ However, past technology integration into clinical workflows has resulted in greater documentation burdens and increased physician burnout, as has been demonstrated from issues in large-scale Electronic Health Records (EHR) systems integration.^57^ The importance of interdisciplinary collaboration to knit people, process, and technology along with patient and family engagement cannot be understated. AI cannot just be assumed to improve clinician workflows; rather, it must be carefully implemented and iteratively evaluated and optimized to ensure that it augments the provision of excellent health care, does not cause new errors and ultimately improves health outcomes for all. Clinical informatics expertise will be critical to ensure seamless and effective integration into patient care “at the elbow”.^58^

Implementing AI into efforts to address the impacts of climate change on pediatric health also requires rigorous evaluation for its effectiveness on improving clinical outcomes equitably. AI systems’ efficacy across clinical applications also cannot be assumed, nor should it be presupposed that they perform equally across disease areas and prediction tasks and are equivalent or superior to clinicians in their applications. Additionally, as climate change’s impacts on pediatric health are most acutely felt amongst children from vulnerable populations, AI systems evaluation must include an equity assessment to ensure that their benefits are being distributed. There is a serious risk of benefits disproportionately improving health and care for populations from already advantaged backgrounds.^59,60^ These populations include those geographically close to health facilities with electronic health record systems that can support embedded AI solutions, those with access to transportation, or with characteristics more likely to be represented in clinical data sets.

### Integrating AI into medical education

Physicians must be prepared to care for patients in the context of climate change. Evidence has demonstrated that while medical students and doctors are aware of the health implications of climate change, they feel unprepared to respond to this crisis.^61^ There is limited research on the quantity and quality of climate change curriculum.^62^ AI creates opportunities for curriculum tracking, development, and support in the medical education space. By collating developing curricula across medical school, graduate medical education, and continuing medical education levels, an AI platform can move climate and health education forward. It can support trainees in learning the latest information and support faculty in staying up to date for teaching purposes. Beyond traditional curriculum, AI may have the potential to offer real-time education by detecting changing patterns (ex: preparing a discharge during a heatwave) and prompting in the moment education (ex: adjusting anticipatory guidance, recommendations on medication storage, etc.).

### Policy implications of AI in health care

As the scope of AI broadens in medicine, health care policy will need to respond. “The National AI Initiative Act of 2020” created a coordinated program across the United States’ federal government to promote AI research and application.^63^ The Centers for Medicare and Medicaid Services (CMS) have developed an “Artificial Intelligence at CMS” webpage to offer guidance on AI development and implementation in the healthcare sector.^64^ However, some raise questions about the benefits that legal and regulatory governance offer versus the benefits of “permissionless innovation”.^65^ With scarce research in this space, there exists ample opportunity to conduct further research exploring the development of AI in healthcare, specifically within the reimbursement landscape, patient privacy, patient data, data storage, equitable treatment of different patient groups, and overall range of applications when it comes to health and climate change. To date there is a large excess of publications reporting test metrics including sensitivity, specificity, positive predictive value, and area under receiver operating characteristic (ROC) or Precision-Recall curves. While such statistics are important in selecting potentially high performing models, they are not able to reliably predict which models will perform well in real clinical environments or positively influence care. Extensive evaluation of usability, user experience and confidence in systems, and clinical trials of impact on patient care and outcomes in representative clinical settings are essential.

## Conclusion and call to action

While still nascent in their development, health information systems and AI applications show great potential to help mitigate the pediatric health burdens that stem from climate change. It is imperative that these solutions are designed ethically and implemented equitably so that their benefits are not disproportionately enjoyed by patients in high income countries or highly resourced areas within LMICs. As the field continues to expand, it is critical to involve researchers from LMICs and develop global networks of research collaboration so that the perspectives of those who are most affected by climate change are amplified. This can also foster local innovation and emphasis on practical, and sustainable solutions. The initial impetus to develop these networks, promote LMIC authorship, and support research in this area falls on researchers and institutions from high income countries.

### Positionality statement

Climate change’s impacts on pediatric health and environmental well-being are felt worldwide, but most acutely impact individuals in resource-denied settings, especially in low- and middle- income countries (LMIC). Before sharing this study’s findings, the authors want to acknowledge their positionality as researchers from highly resourced US and German institutions, with access to resources and education that the many communities most impacted by climate change do not have. Authors bring extensive knowledge of and lived experience in global health informatics in LMICs as well as climate change and health, however none have academic institutional homes based in an LMIC, largely due to a lack of international research community on this subject matter that our authors could draw a connection from. It is the goal of the authors to highlight the importance of these issues and support our colleagues in highly impacted countries in their research and practice to address the effects of climate change.

## Appendix

Final Query: (“Intelligence, Artificial” OR “Machine Intelligence” OR “Intelligence, Machine” OR “Artificial Intelligence” OR “Machine Learning” OR “Deep Learning” OR “Supervised Machine Learning” OR “Support Vector Machine” OR “Unsupervised Machine Learning” OR “Computer Heuristics” OR “Expert Systems” OR “Fuzzy Logic” OR “Natural Language Processing” OR “Neural Networks, Computer” OR “Robotics” OR “Sentiment Analysis”) AND (“Pediatric Medicine” OR “Child Services, Health” OR “Health Services, Child” OR “Child Health Service” OR “Health Service, Child” OR “Service, Child Health” OR “Services, Child Health” OR “Infant Health Services” OR “Infant Services, Health” OR “Health Services, Infant” OR “Health Service, Infant” OR “Infant Health Service” OR “Service, Infant Health” OR “Services, Infant Health” OR “Pediatrics” OR “Adolescent Medicine” OR “Pediatric Emergency Medicine” OR “Child” OR “Child Health” OR “Health, Child” OR “Children’s Health” OR “Health, Children’s” OR “ Child Well Being” OR “Well Being, Child” OR “Well-Being, Child” OR “Child Wellbeing” OR “Wellbeing, Child”) AND (“Climate Change” OR “Change, Climate” OR “Changes, Climate” OR “Climate Changes”).
